# Chemical Composition, Antioxidant, and Antibacterial Activities of *Mezilaurus duckei* (Lauraceae)

**DOI:** 10.1002/cbdv.202500826

**Published:** 2025-09-09

**Authors:** Bruna S. Moroto, Valéria S. Gonçalves, David J. Machate, Talita V. Freire, Érica L. Santos, Flávio M. Alves, Ana C. Micheletti, Nídia C. Yoshida

**Affiliations:** ^1^ Instituto De Biociências Universidade Federal de Mato Grosso Do Sul Campo Grande Brazil; ^2^ Instituto De Química Universidade Federal de Mato Grosso Do Sul Campo Grande Brazil

**Keywords:** Amazon rainforest, bioactive compounds, flavonoid, kaempferol, rubrenolide

## Abstract

*Mezilaurus duckei*, a Brazilian endemic tree species found exclusively in the Amazon Rainforest, is primarily exploited for timber in construction. Due to its endangered status, this study aimed to investigate the chemical profile and biological properties of the ethanolic extract and its phases derived from *M. duckei* leaves. Chemical profiling using high‐performance liquid chromatography‐tandem mass spectrometry revealed flavonoids, including glycosylated kaempferol and its derivatives, as well as γ‐butyrolactones, as the major constituents. Additional chromatographic separations led to the isolation of kaempferol, reported for the first time in the *Mezilaurus* genus, and rubrenolide, identified for the first time in *M. duckei*. Antioxidant activity, assessed using the 2,2‐diphenyl‐1‐picrylhydrazyl method, demonstrated significant free radical scavenging properties of the ethanolic extract (half‐maximal inhibitory concentration [IC_50_] = 1.43 µg/mL) and the ethyl acetate phase (IC_50_ = 1.28 µg/mL). Antimicrobial activity, evaluated via the microdilution method, revealed promising activity of the dichloromethane fraction against *S. aureus* and *E. coli* (minimum inhibitory concentration values of 185.5 and 375 µg/mL, respectively). Toxicity assessment using the *Artemia salina* lethality assay confirmed the non‐toxic nature of the ethanolic extract (50% lethal dose > 1000 µg/mL). These results highlight the potential of *M. duckei* as a source of bioactive compounds, undescoring their relevance for further applications.

## Graphical abstract

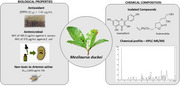

## 1 Introduction

The Lauraceae family comprises 58 genera and 3,384 species worldwide [1], with 27 genera and 480 species occurring in Brazil [2], many of which have significant food and medicinal uses [3, 4, 5, 6]. Notable examples of food species include avocado (*Persea americana* Mill.), cinnamon (*Cinnamomum verum* J. Presl), among others [7, 8, 9, 10]. However, some species within the *Aniba*, *Nectandra*, *Ocotea*, and *Mezilaurus* genera are utilized in carpentry, timber production, and civil construction, and are also widely used for essential oil extraction [11, 12, 13, 14, 15].

*Mezilaurus* is a Neotropical genus with about 17 species of trees or shrubs, ranging from Colombia and Suriname to Bolivia and Southeastern Brazil. Three‐fifths (ca. 60%) of its species are endemic to the Amazonian Forests domain, one‐fifth are exclusive to the Brazilian Atlantic forest, while the remaining one‐fifth are typical to the Brazilian savannas, that is, Cerrado [2, 16, 17, 18].

Due to the appreciable quality and durability of the wood from some *Mezilaurus* spp., it is widely used for carpentry and for boat construction in the Amazon region, which is extensively traversed by numerous navigable rivers [19]. In addition, the traditional medicinal and pharmacological uses of *Mezilaurus* species are supported by studies demonstrating the bioactive properties of essential oils and extracts obtained from *M. crassiramea* (Meisn.) Taub. ex Mez, *M. itauba* (Meisn.) Taub. ex Mez, and *M. duckei* van der Werff. These extracts exhibit antibacterial, antiprotozoal, antioxidant, and anticancer activities against various cancer cell lines, including breast (MCF‐7 and MDA‐MB‐231), colon (HT‐29), prostate (PC‐3), renal (786‐0), and leukemia (HL‐60, GI_50_ = 0.21 µM) [20, 21, 22, 23]. Moreover, the γ‐lactone rubrenolide, described for the first time as a natural product from the leaves extract of *M. crassiramea*, demonstrated significant activity against cancer cell lines, such as human melanoma (UACC‐62, GI_50_ = 3.3 µg/mL), breast adenocarcinoma (MCF‐7, GI_50_ = 3.7 µg/mL), colon adenocarcinoma (HT‐29, GI_50_ = 5.1 µg/mL), prostate adenocarcinoma (PC‐3, GI_50_ = 9.9 µg/mL), kidney adenocarcinoma (786‐0, GI_50_ = 18.8 µg/mL), and ovarian adenocarcinoma (CI/ADR‐RES, GI_50_ = 22.6 µg/mL). Other γ‐lactone derivatives, such as 3’‐acetylrubrenolide and 2’,3’‐diacetylrubrenolide, exhibited moderate activity [23].

Although some *Mezilaurus* species are highly valued for their wood in carpentry and boat construction, and are known to produce extracts, essential oils, and isolated compounds with notable bioactive properties, most species within this genus remain largely unexplored regarding their chemical composition and pharmacological potential. Therefore, studies are needed to identify bioactive compounds that could enable alternative applications, allowing sustainable use of these species without the destructive practice associated with timber harvesting.

Moreover, *M. duckei* is popularly known by several vernacular names, including “itaúba,” “itaúba‐avocado,” “laurel‐itaúba‐avocado,” or “ubá”. The diversity of common names, together with the morphological similarity between *Mezilaurus* species, often leads to confusion among different botanical species. *Mezilaurus duckei* is a large and rare tree for which chemical and biological investigations remain scarce in the literature, with only one previous report describing the chemical composition of the essential oil from its leaves [24]. The species is endemic to the state of Amazonas in Brazil, with a single recorded occurrence in Colombia [1, 2]. Associated with the *Mezilaurus* spp. value for timber exploitation, the *M. duckei* is currently considered at risk of extinction [25].

Therefore, this study aimed to determine the chemical profile, antioxidant activity, antimicrobial properties, and toxicity effects of the ethanolic leaf extract and phases of *M. duckei*.

## 2 Results and Discussion

### 2.1 Chemical Profile of the *M. duckei* Leaves Extract

**FIGURE 1:**
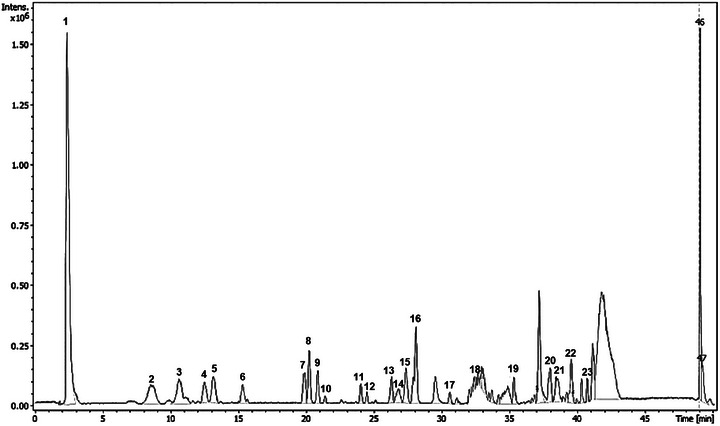
Base peak chromatogram (BPC, positive mode) of *Mezilaurus duckei* leaves methanolic extract.

The chemical profile of the *M. duckei* leaf extract was determined using high‐performance liquid chromatography with diode‐array detection and tandem mass spectrometry (HPLC‐DAD‐MS/MS) analysis, which revealed the presence of 23 major chromatographic peaks (Figure 1).

Through the analysis of the data set, it was possible to annotate 18 compounds, belonging to flavonoid, neolignan, alkaloid, γ‐butyrolactone, phytosterols, and pheophorbide classes. Eleven compounds (**2**, **4**, **8**, **13–16**, **19**, **20**, **22**, and **23**) were identified or tentatively characterized based on retention time (RT), online UV, and HRESIMS data, including mass fragmentation patterns. These data were compared with those of authentic standards, published data, or both (Table 1).

**TABLE 1:** Chemical constituents annotated in the methanolic extract analysis of *Mezilaurus duckei* by high‐performance liquid chromatography with diode‐array detection and tandem mass spectrometry (HPLC‐DAD‐MS/MS).

Peak	RT [min]	Positive *m/z*	MS/MS (intensity)	UV [nm]	Molecular formula [ion molecule]	Metabolite class/tentative assignment
**1**	2.4	319.1869	—	254, 330	C_17_H_28_O_4_ [M+Na]^+^	Unknown
**2**	8.6	619.1275	303.0501 (100)	254, 330	C_26_H_28_O_16_ [M+Na]^+^	Flavonoid/Quercetin‐3‐*O*‐*β*‐D‐apiofuranosyl‐(1→2)‐*O*‐*β*‐D‐galactopyranoside
**3**	10.6	603.1319	287.0550 (100)	254, 330	C_26_H_28_O_15_ [M+Na]^+^	Flavonoid
**4**	12.5	471.0896	287.0552 (50.0)	254, 330	C_21_H_20_O_11_ [M+Na]^+^	Flavonoid/Kaempferol 3‐*O*‐*β‐*D‐glucopyranoside
**5**	13.2	471.0894	287.0547 (70.9)	245, 330	C_21_H_20_O_11_ [M+Na]^+^	Flavonoid
**6**	15.3	587.1356	287.0546 (68.6)	254, 330	C_26_H_28_O_14_ [M+Na]^+^	Flavonoid
**7**	19.9	355.1159	—	330	C_18_H_20_O_6_ [M+Na]^+^	Neolignan
**8**	20.2	287.0554	—	254, 330	C_18_H_36_O_5_ [M+H]^+^	Flavonoid/Kaempferol^*^ ^ST^
**9**	20.9	365.1064	—	—	C_14_H_21_O_11_ [M+Na]^+^	Unknown
**10**	21.4	362.3275	—	—	C_21_H_40_O_5_ [M+H]^+^	Unknown
**11**	24.1	302.3066	—	—	C_18_H_40_NO_2_ [M+H]^+^	Alkaloid
**12**	24.5	346.3322	—	—	C_20_H_44_NO_3_ [M+H]^+^	Alkaloid
**13**	26.3	321.2046	—	330	C_17_H_30_O_4_ [M+Na]^+^	γ‐butyrolactone/Rubrenolide^*^ ^ST^
**14**	26.8	747.1699	—	254, 330	C_39_H_32_O_14_ [M+Na]^+^	Flavonoid/Kaempferol dicoumaroyl‐rhamnopyranoside derivative
**15**	27.4	747.1716	—	254, 330	C_39_H_32_O_14_ [M+Na]^+^	Flavonoid/Kaempferol‐3‐*O‐*α‐L‐[2‐(*Z*)‐*p*‐cumaroil‐4‐(*E*)‐*p*‐coumaroyl] rhamnopyranoside
**16**	28.1	323.2201	—	330	C_17_H_32_O_4_ [M+Na]^+^	γ‐butyrolactone/Dihydrorubrenolide
**17**	30.6	677.3735	—	—	C_33_H_57_O_14_ [M+Na]^+^	Unknown
**18**	32.4	429.3229	—	—	C_24_H_45_O_6_ [M+H]^+^	Steroid
**19**	35.3	381.2982	—	—	C_23_H_41_O_4_ [M+Na]^+^	γ‐butyrolactone/γ‐butyrolactone derivative
**20**	38.0	599.4287	—	—	C_35_H_60_O_6_ [M+Na]^+^	Steroid/Sitosterol 3‐*O*‐*β*‐D‐glucopyranoside^*^ ^ST^
**21**	38.5	609.2713	—	—	C_35_H_37_N_4_O_6_ [M+H]^+^	Alkaloid
**22**	39.4	469.3618	—	—	C_29_H_50_O_3_ [M+Na]^+^	Terpenoid
**23**	41.8	593.2770	—	—	C_60_H_75_N_2_O_3_ [M+H]+	Pheophorbide/Pheophorbide a5^*^ ^ST^

*^ST^: confirmed by comparison of authentic standard; RT: retention time; main MS/MS fragment obtained by positive ion mode.

**FIGURE 2:**
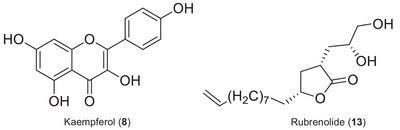
Secondary metabolites isolated from *M. duckei* leaves.

Additional chromatographic separation was performed on the dichloromethane phase to afford the flavonoid kaempferol (**8**) and a γ‐butyrolactone rubrenolide (**13**) (Figure 2). Subsequently, these compounds were characterized using nuclear magnetic resonance (NMR), ultraviolet (UV), and high‐resolution electrospray ionization MS (HRESIMS) data (Supporting Information). These compounds are being reported for the first time in *M. duckei*, and kaempferol is being described for the first time in the *Mezilaurus* genus. However, due to the limited quantity of isolated compounds obtained, they were not subjected to the bioassays.

In the chemical profile of *M. duckei*, it is noteworthy the presence of flavonoids as main compounds, corresponding to peaks **2**–**6, 8, 14**, and **15**, with UV absorption bands observed between 254 and 330 nm.

**Peak 2** exhibited an ion peak at *m/z* 619.1275, corresponding to the proposed molecular formula C_26_H_28_O_16_ [M+Na]⁺, along with a characteristic fragment ion at *m/z* 303, indicative of the flavonoid quercetin‐3‐*O*‐β‐D‐apiofuranosyl‐(1→2)‐O‐β‐D‐galactoside. [Correction added on September 19, 2025, after first online publication: Peak 4 was corrected to Peak 2 in the preceding sentence.]. This compound has previously been reported in *Machilus philippinensis* Merr. (Lauraceae), and other quercetin derivatives have been widely described in Lauraceae species, such as *Laurus novocanariensis* Rivas Mart., *Apollonias barbujana* (Cav.) Bornm., *Ocotea foetens* Aiton Baill., and *Persea indica* (L.) Spreng [26]. Furthermore, this glycosylated flavonoid has also been identified in the Convolvulaceae family, particularly in *Cuscuta chinensis* Lam., *C. australis* R. Br., and *C. japonica* Choisy—species commonly used in traditional Chinese medicine for treating male infertility, kidney tonification, and other therapeutic purposes [27, 28, 29, 30, 31]. Plant extracts rich in flavonoids, including quercetin‐3‐*O*‐β‐D‐apiofuranosyl‐(1→2)‐O‐β‐D‐galactoside, have been associated with diverse bioactivities, such as anticancer, antiviral, antinociceptive, and anti‐inflammatory effects [29].

It was noteworthy that the extract from leaves of *M. duckei* stood out as a kaempferol aglycone‐rich extract, exhibiting several peaks corresponding to flavonoids with a suggested kaempferol aglycone, based on the *m/z* 287 fragment observed in compounds **3–6** and **14** and **15**. Except for compound **2**, all other peaks attributed to flavonoids displayed similar *m/z* 287 fragments, suggesting the presence of the same kaempferol aglycone. Previous reports have already described the presence of this flavonoid and its glycosides in species of the Lauraceae family, particularly in *Lindera neesiana*, with biological properties such as antioxidant activity, lipase inhibitory effects, and potent antimicrobial activity [32].

**Peak 8** (RT 20.2 min) presented *m/z* 287.0554, corresponding to the [M+H]^+^ ion of kaempferol, with molecular formula C_15_H_10_O_6_, which was also isolated from *M. duckei* and identified by NMR analysis. The kaempferol compound is one of the major secondary metabolite found in high amount in plant kingdom, and it is found in significant amounts in several different plants, such as *Capparis spinosa* (2590 mg/kg), *Crocus sativus* (2050 mg/kg), *Allium cepa* (832 mg/kg), *Allium fistulosum* (832 mg/kg), *Carica papaya* (453 mg/kg), *Cucurbita maxima* (372 mg/kg), *Sauropus androgynous* (323.5 mg/kg), *Phaeomeria speciosa* (286 mg/kg), *Daucus carota* (140 mg/kg), *Camelia sinensis* (118 mg/kg), *Raphanus sativus* (38.5 mg/kg), and others [33, 34]. Kaempferol has a high affinity for fatty acids and is absorbed in the small intestine. It offers various health benefits, including antioxidant, anti‐aging, anticancer, anti‐inflammatory, anti‐diabetic, anti‐pathogenic, antiprotozoal, antifungal, and antibacterial effects [34, 35, 36, 37].

**Peak 13** (RT 26.3 min) showed *m/z* 321.2069, compatible with the chemical formula C_19_H_29_O_4_ [M+Na]^+^, corresponding to the γ‐butyrolactone rubrenolide, isolated from *M. duckei* and identified by NMR analysis, and previously described to Lauraceae species such as *Sextonia rubra* (Mez) van der Werff (with its synonymies *Ocotea rubra* Mez and *Nectandra rubra* (Mez) C.K.Allen), *Aiouea trinervis* Meisn., and *Mezilaurus crassiramea* (Meisn.) Taub. ex Mez [2, 38, 39, 40]. Butanolide compounds from *M. crassiramea* demonstrated anticancer activity against several cell lines (MCF‐7 and MDA‐MB‐231 (breast), HT‐29 (colon), PC‐3 (prostate), 786‐0 (renal), HL‐60 (leukemia) [20], antiprotozoal, and anti‐trypanosomal activities [38]. In addition, peaks **16** and **19** showed ions with *m/z* at 323.2201 (RT 28.1 min) and 381.2982 (35.3 min), corresponding to C_17_H_32_O_4_ [M+Na]^+^ and C_23_H_41_O_4_ [M+Na]^+^, respectively, and were also annotated as γ‐butyrolactones derivatives.

**Peak 18** (RT 32.4 min.) showed *m/z* 429.3229, with the proposed molecular formula C_24_H_45_O_6_ [M+H]^+^ (4.7 ppm error), referring to three possible different steroid compounds, being sepesteonol, stigmastan‐3,6‐dione, or (3*β*)‐3‐hydroxyestigmast‐5‐en‐7‐one. Belonging to the same class, the compound corresponding to **peak 20** (RT 38.0 min) with the molecular formula C_35_H_60_O_6_ could be attributed to the sitosterol 3‐*O‐β*‐D‐glucopyranoside, which is commonly found in plants.

**Peak 23** (RT 41.8 min) showed an *m/z* of 593.2770, corresponding to the molecular formula C_60_H_75_N_2_O_3_, which is compatible with pheophorbide a5. Both compounds **20** and **23** were identified based on reference compounds.

Fourteen compounds of the extract from *M. duckei* leaves remained unidentified (**1**, **3**, **5–7**, **9–12**, **17**, **18**, **21**, **22,** and **37**), presenting opportunities for further research. These unknown compounds, annotated through their distinct mass data, point to the potential existence of uncommon metabolites.

### 2.2 Antibacterial, Antioxidant, and Toxicity Properties of *M. duckei* Leaves Extract and Phases

The extract of *M. duckei* leaves and its phases were evaluated against bacterial strains of *Staphylococcus aureus* (Gram‐positive) and *Escherichia coli* (Gram‐negative), and the results are summarized in Table 2. The biological activities observed in this study are likely attributable to synergistic effects among constituents, as well as to the individual contributions of compounds such as kaempferol (**8**) and rubrenolide (**13**), as previously reported [34, 35, 36, 37, 38]. The dichloromethane fraction exhibited moderate antibacterial activity, with minimum inhibitory concentrations (MICs) of 185.5 µg/mL against *S. aureus* and 375 µg/mL against *E. coli*. In contrast, the ethanolic extract and the hexane, ethyl acetate, and hydromethanolic fractions showed weak effects against these strains. According to the literature, MIC values are classified as active when <100 µg/mL, moderate when 100< MIC ≤ 625 µg/mL, and weak or negligible when >625 µg/mL [41, 42]. Although the activities observed here do not yet justify practical applications, further in‐depth investigations are warranted, particularly on the dichloromethane fraction, to identify individual or synergistic compounds responsible for the moderate antibacterial effects, especially against Gram‐negative bacteria. This is of considerable interest given the well‐recognized challenge of discovering bioactive compounds effective against this bacterial group.

**TABLE 2:** Minimum inhibitory concentration (MIC) values of the *Mezilaurus duckei* ethanolic extract and its respective phases against bacterial strains.

Sample	*Staphylococcus aureus*	*Escherichia coli*
	MIC (µg/mL)	
Ethanolic extract	≥750	≥750
Hexane phase	≥1500	≥750
Dichloromethane phase	185.5	375
Ethyl acetate phase	≥1500	≥750
Hydromethanolic phase	≥1500	≥750
Gentamicin	≤0.5	≤0.5

Moreover, Gram‐negative bacterial genera, including *Escherichia, Proteus, Enterobacter, Klebsiella, Citrobacter, Yersinia, Shigella*, and *Salmonella*, are commonly associated with hospital‐acquired infections and various human diseases such as urinary tract infections, pneumonia, diarrhea, meningitis, sepsis, and endotoxic shock [43, 44]. The bacterial resistance of Gram‐negative strains is largely attributed to the structural complexity of their outer envelope, which consists of lipopolysaccharides with endotoxic effects and transmembrane proteins (such as porins). These components selectively regulate the passage of small molecules (e.g., amino acids, small saccharides) into the bacterial cell, further complicating treatment [45].

The evaluation of the antioxidant capacity, assessed using the 2,2‐diphenyl‐1‐picrylhydrazyl (DPPH) method, demonstrated a strong activity for the ethanolic extract (half‐maximal inhibitory concentration [IC_50_] = 1.43 µg/mL) and the ethyl acetate phase (IC_50_ = 1.28 µg/mL), compared to the control caffeic acid, with an IC_50_ of 1.52 µg/mL (Table 3). This remarkable antioxidant activity exhibited by the extract of *M. duckei* leaves and the ethyl acetate phase is likely due to the predominance of flavonoids as key constituents. Flavonoids, which are widely distributed across the plant kingdom, offer numerous health benefits, including antioxidant, anti‐inflammatory, anticancer, anti‐cardiovascular, antiviral, antibacterial, antifungal, antiprotozoal, and antimicrobial effects [46, 47]. Among these flavonoids, the secondary metabolites quercetin and kaempferol—identified in this study, with kaempferol being reported for the first time in *Mezilaurus*—stand out in this context for their well‐established health benefits and strong antioxidant properties [33, 48]. Their ability to scavenge free radicals and prevent oxidative damage has drawn significant attention from the pharmaceutical and healthcare industries. However, although their antioxidant activity is prominent, their antimicrobial properties are equally important, as plants often synthesize flavonoids as a defense response to microbial infection, and these compounds demonstrate potent antimicrobial action against a wide range of pathogenic microorganisms. Antibacterial effects involve diverse mechanisms, such as disruption of cell membranes, inhibition of DNA gyrase, and suppression of fatty acid biosynthesis. Beyond direct antimicrobial activity, some flavonoids can inhibit bacterial virulence factors, prevent biofilm formation, and even reverse antibiotic resistance, enhancing the efficacy of conventional drugs [49]. In this context, kaempferol and quercetin stand out for their antibacterial properties against pathogens, including *E. coli*, methicillin‐resistant *S. aureus* (MRSA), *Micrococcus luteus*, and *Pseudomonas aeruginosa* [34].

**TABLE 3:** 2,2‐diphenyl‐1‐picrylhydrazyl (DPPH) antioxidant assay of the *Mezilaurus duckei* leaves extract and phases.

Sample	IC_50_ (µg/mL) ± SD
Ethanolic extract	1.43 ± 0.33
Hexane phase	3.62 ± 0.12
Dichloromethane phase	2.17 ± 0.20
Ethyl acetate phase	1.28 ± 0.34
Hydromethanolic phase	2.47 ± 0.32
Caffeic acid	1.52 ± 0.56

Previous studies have reported antioxidant activity in extracts from the leaves and branches of *M. duckei*, with IC_50_ values of 23.25 and 13.49 µg/mL, respectively [22]. The discrepancies observed between the present and prior investigations may be attributed to factors such as seasonal variations, geographical origin of the plant material, and the employment of different experimental controls (quercetin) in previous work [22]. Furthermore, the concentration of bioactive constituents is known to vary according to the collection site, which can substantially impact the biological activities evaluated, including the antimicrobial and antioxidant properties described herein. Recent findings by Sin et al. showed marked variation in flavonoid levels even within genetically similar plants. Notably, kaempferol‐3‐*O*‐glucuronide and quercetin‐3‐*O*‐rhamnoside were more abundant in high‐flavonoid genetic clusters, with environmental factors and stress adaptability identified as key drivers of this accumulation [50].

Additionally, γ‐butyrolactones have been reported to exhibit anti‐inflammatory and anticancer properties, which are often linked to the antioxidant activity. This class of compounds, which includes rubrenolide, is frequently found in Lauraceae species with notable bioactive potential, such as *Machilus zuihoensis* var. *mushaensis* and *M. japonica* var. *kusanoi* (Lauraceae) [23, 51, 52].

Finally, in the toxicity assay using *Artemia salina*, a sample is considered active if it exhibits a 50% lethal dose (LD_50_) value below 1000 µg/mL [53]. Our findings indicate that the extract of *M. duckei* leaves is non‐toxic, as its LD_50_ value exceeded 1000 µg/mL.

## 3 Conclusions

The chemical profile of the ethanolic extract of *M. duckei* leaves revealed the presence of compounds belonging to the flavonoid, terpene, and γ‐butyrolactone classes. The dichloromethane phase exhibited moderate antibacterial activity against Gram‐positive bacteria (*S. aureus*, MIC = 185.5 µg/mL) and Gram‐negative bacteria (*E. coli*, MIC = 375 µg/mL). Additionally, the ethanolic extract of *M. duckei* showed no toxic effects against *Artemia salina*. In contrast, both the extract and its ethyl acetate phase demonstrated significant antioxidant activity against the DPPH radical, with IC_50_ values of 1.43 and 1.28 µg/mL, respectively. Notably, this study is the first to report the presence of the flavonoid kaempferol in the genus *Mezilaurus*, specifically in *M. duckei*, for which the γ‐butyrolactone rubrenolide is described for the first time.

These findings underscore the promising bioactive potential of *M. duckei*, an underexplored Amazonian species with restricted distribution and currently at risk of extinction due to timber exploitation. The demonstrated antimicrobial and antioxidant activities of its leaf extracts and fractions support its sustainable use as a source of biologically active compounds and justify further pharmacological and biotechnological investigations.

## 4 Experimental

### 4.1 General Experimental Procedures

DPPH and caffeic acid were purchased from Sigma‐Aldrich (USA). Solvents used in the extraction and fractionation procedures were obtained from Dinamica Química (Brazil). ^1^H and ^13^C NMR spectra were obtained in CDCl_3_ and acetone‐d6 (Cambridge Isotope Laboratories) on a Bruker DPX‐300 spectrometer (Bruker) operating at 300.13 MHz (^1^H)/75.47 MHz (^13^C). [Correction added on September 19, 2025, after first online publication: “CD3OD” was corrected to “acetone‐d6” in the preceding sentence.]. Column chromatography (CC) procedures were performed on silica gel 60 (70−230 mesh, Merck, Germany) and Sephadex LH‐20 (Sigma‐Aldrich, USA). TLC silica gel GF_254_ plates were purchased from Merck (Germany). HPLC‐DAD‐MS/MS was performed using an LC‐DAD‐HRESIMS system equipped with a SIL‐20A autosampler, a DGU‐20A3r vacuum degasser, a thermostated CTO‐20A column compartment, and an LC20AD pump, coupled to an SPD‐M20A DAD (all Shimadzu, Japan) and a micrOTOF Q‐III high‐resolution time‐of‐flight mass spectrometer (Bruker Daltonics, USA) with an electrospray ionization (ESI) ion source, operating in positive ion mode (120−1200 Da and collision energy 45−65 V).  The reference bacterial strains *S. aureus* (NEWP0023) and *E. coli* (NEWP0022) were purchased from NEWPROV Company (Brazil).

### 4.2 *M. duckei* Leaves Collection and Preparation

Fresh leaves of *M. duckei* (236.30 g) were collected in August 2018 from Itacoatiara, Amazonas state, Brazil (2°27'24.60''S, 58°38'53.10''W). The plant material was taxonomically identified by botanist Dr. Flavio Macedo Alves, and a voucher specimen was deposited at the herbarium of the Instituto Nacional de Pesquisas Amazônicas (INPA) under the number 281336. Collection and research were authorized under Brazil's biodiversity license #A872A67. The leaves were shade‐dried at room temperature, ground using a mortar and pestle, and sieved to obtain a fine powder. The resulting material was stored in a hermetically sealed amber glass container at –20 °C until further analysis.

### 4.3 HPLC‐DAD‐MS/MS Chemical Profiling

Dried leaves of *M. duckei* (25 mg) were placed in 2 mL microtubes and subjected to ultrasound‐assisted extraction using 1 mL of HPLC‐grade methanol for 12 min. The samples were then centrifuged at 10 000 rpm for 4 min, and the resulting supernatant was collected and evaporated to dryness at room temperature.

Aliquot of the extract (10 µL of methanolic solution at 1.0 mg/mL) was injected into the HPLC‐DAD‐MS/MS. The eluent system used was water and methanol (both containing 0.1% formic acid), ranging from 3% to 80% of methanol (v/v), totaling 45 min of analysis, with a flow of 0.2 mL/min and positive ionization mode.

The annotation of the compounds presents in the chemical profile, as well as the proposition of the molecular formulas (considering only error values < 5 ppm), were performed through data analysis using the Compass DataAnalysis 4.2 software (Bruker, USA) and through a database assembled from SciFinder (Chemical Abstracts Service–CAS, USA) data platform, in which compounds already registered for the Lauraceae family were searched. For data comparison, the RT, [M‐H]^+^ ions, and their main molecular ions, UV absorption spectra, *m/z* values, and data obtained from isolated compounds and in the literature were analyzed.

### 4.4 Extraction and Compounds Isolation From *M. duckei* Leaves Ethanolic Extract

A powdered sample of *M. duckei* leaves (236.30 g) was subjected to exhaustive maceration with ethanol (800 mL) at 25 °C. Every 48 h, the supernatant was collected and concentrated under reduced pressure, then stored in hermetically sealed amber glass bottles at –20 °C. This procedure was repeated over a 28‐day period to yield 13.89 g of dried extract. This extract was subsequently subjected to liquid–liquid partitioning, affording the following solvent fractions: hexane, dichloromethane, ethyl acetate, and hydromethanolic. The phases were analyzed by thin‐layer chromatography (TLC) on silica gel GF_254_ plates, and the mobile phase consisted of chloroform:methanol (8:2, v/v). Chromatographic development was carried out using Korte's reagent (*p*‐hydroxylamine/ferric chloride), which enables the visualization of esterified compounds such as lactones, a class of metabolites of particular interest in this study. A distinct pink spot, indicative of lactone presence, was observed in the dichloromethane phase. Based on this observation, the dichloromethane phase (1.59 g) was selected for subsequent chromatographic fractionation on a Sephadex LH‐20 gel column using a chloroform:methanol (6:4, v/v) eluent system (column dimensions: 2.5 cm in diameter and 31 cm in height). Fourteen fractions (A–N) were collected and analyzed by TLC in the same conditions previously described. Fraction F (27 mg) was further purified by preparative TLC on silica gel GF_254_ using chloroform:methanol (9:1, v/v) as the eluent, affording rubrenolide (2 mg, RF = 0.6). Fraction M yielded kaempferol (10 mg). The isolated compounds were characterized by nuclear magnetic resonance (NMR) spectroscopy, recorded on a Bruker DPX‐300 MHz spectrometer using deuterated acetone and chloroform (acetone‐d_6_ and CDCl_3_) as solvents.

Kaempferol (**8**): ^1^H NMR (300 MHz, Acetone‐d_6_, J in Hz): 6.26 d (J = 1.8), 6.53 d (J = 1.8), 8.14 d (J = 8.5), 7.00 d (J = 8.5), 7.00 d (J = 8.5), 8,14 d (J = 8.5).

Rubrenolide (**13**): ^1^H NMR (300 MHz, CDCl_3_, J in Hz): 2.83–2.94 m, 2.53 ddd (J = 12.3, 8.4, 5.4), 1.54–1.62 m, 4.31–4.42 m, 1.71–1.81 m, 1.56–1.66 m, 1.42–1.52 m, 1.32–1.42 m, 1.25–1.40 sl, 1.25–1.40 sl, 1.25–1.40 sl, 1.25–1.40 m, 1.25–1.40 sl, 2.00 dd (J = 14.0, 6.6), 5.75 ddt (J = 16.8, 11.2, 6.6), 4.95 dL (J = 16.8), 4.89 dL (J = 11.2), 1.90–2.00 m, 1.55–1.61 m, 3.65–3.75 m, 3.59 dL (J = 10.8), 3.44 dd (J = 0.8, 6.3). ^13^C NMR: 180.0, 38.7, 35.7, 79.9, 35.3, 25.2, 29.3, 29.0, 29.3, 28.8, 29.3, 33.7, 139.1, 114.1, 33.7, 70.2, 66.6. HRESIMS: *m/z* 298.2140 (calc. 298.2140, C_17_H_30_O_4_).

### 4.5 Antimicrobial Activity and Determination of MIC

The antibacterial activity of the samples was evaluated by the broth microdilution assay according to CLSI (Clinical Laboratory Standards Institute) standards [54], in which strains of *S. aureus* (Gram‐positive) and *E. coli* (Gram‐negative) were tested.

Five samples were evaluated in this study: the crude ethanolic extract of *M. duckei* leaves and its hexane, dichloromethane, ethyl acetate, and hydromethanolic phases. The samples (6 mg/mL), in DMSO, were applied to 96‐well microplates, previously filled with 100 µL of Mueller‐Hinton broth per well. Gentamicin was used as a positive control, at a concentration of 60 to 0.5 µg/mL, from the initial to final, respectively; only culture medium was used as a negative control. All experiments were conducted in triplicate.

The plates were incubated at 36°C for 18 h. After that, 20 µL of an aqueous solution (0.5%) of triphenyltetrazolium chloride (TTC) was added to each well and the plates were incubated again at 36°C for 2 h. The color changes of TTC from colorless to red indicate active cell metabolism. Therefore, our analyses do not show color changes, which means the sample is weak in the MIC, defined as the lowest concentration in each sample capable of inhibiting bacterial growth [54].

### 4.6 Antioxidant Activity by DPPH Radical Assay

The antioxidant activity of the extract and its phases (hexane, dichloromethane, ethyl acetate, and hydromethanolic phases) was evaluated using the DPPH free radical scavenging assay, following the method described by Yamaguchi et al. [54]. Antioxidant activity was assessed based on the ability of the antioxidant sample to reduce DPPH, a process monitored by the decrease in absorbance of the DPPH solution [55, 56].

A DPPH stock solution (10 mg) was prepared in 50 mL of ethanol. Samples were dissolved in ethanol at a concentration of 200 µg/mL and applied to 96‐well microplates previously loaded with 100 µL of ethanol. In the first row of wells, 200 µL of each sample was added individually in triplicate. Serial dilutions were then performed across the subsequent wells, except for the last row, which served as the blank.

The final concentrations tested ranged from 200 to 3.125 µg/mL. Caffeic acid was used as a positive control due to its well‐documented antioxidant, antimicrobial, and anti‐inflammatory properties widely established in the literature [57, 58, 59, 60]. The microplates were covered with aluminum foil and incubated at 30 °C for 30 min in the dark. Absorbance readings were taken at 517 nm using a SpectraMax Plus 384 microplate reader (Molecular Devices, USA).

### 4.7 Toxicity Test on *Artemia Salina*

The brine shrimp toxicity assay was evaluated according to Meyer and collaborators [58]. Eggs of the microcrustacean *Artemia salina* were prepared in saline solution, at a concentration of 38 g of sea salt per litre of distilled water and pH between 7 and 8, in an environment with artificial lighting and constant oxygenation. Eggs hatch in the solution for 24 h. The analyses were conducted in triplicate, testing the ethanolic extract of *M. duckei* leaves at concentrations of 1000, 500, 250, and 125 µg/mL.

The extract was dissolved in dimethyl sulfoxide (DMSO) to achieve the desired concentrations, and 7 mL of each solution was used to expose 10 *Artemia salina* larvae per assay. Positive controls consisted of saline water containing 400 µL of DMSO and 20 mg of quinidine sulfate, while negative controls contained saline solution with 400 µL of DMSO only. After 24 h, the number of surviving and dead larvae was recorded, and LD_50_ values (µg/mL) were calculated using Probit analysis. Extracts presenting LD_50_ values below 1000 µg/mL were considered potentially active [53, 61].

## Author Contributions

**Bruna S. Moroto**: investigation, formal analysis, and data curation. **Valéria S. Gonçalves**: investigation, formal analysis, and methodology. **David J. Machate**: writing – original draft and writing – review and editing. **Talita V. Freire**: visualization and writing – review and editing. **Érica L. Santos**: investigation, visualization, and writing – review and editing. **Flávio M. Alves**: conceptualization of the topic and writing – review and editing. **Ana C. Micheletti**: formal analysis and writing – review & editing. **Nídia C. Yoshida**: conceptualization the topic, investigation, formal analysis, writing – original draft, visualization, data curation, supervision, methodology, writing – review and editing, funding acquisition, and project administration. We have read and approved the final version to the published the manuscript.

## Conflicts of Interest

The authors declare no conflict of interest.

## Supporting information




**Supporting File 1**:cbdv70453‐sup‐0001‐SupMat.docx

## Data Availability

The authors have nothing to report.
