# Supplementary material for: Chemical Composition, Antioxidant, and Antibacterial Activities of Mezilaurus duckei (Lauraceae)
Source: Chem Biodivers. 2025 Sep 9;22(12):e00826. doi: 10.1002/cbdv.202500826 (PMC12715993; doi:10.1002/cbdv.202500826)
Supplement: Supplementary file 1 — Supporting File 1:cbdv70453‐sup‐0001‐SupMat.docx [file CBDV-22-e00826-s001.pdf]

SUPPLEMENTARY MATERIAL

Chemical Composition, Antioxidant and Antibacterial Activities of *Mezilaurus duckei* (Lauraceae)

Bruna S. Moroto,[a] Valéria S. Gonçalves,[b] David J. Machate,[b] Talita V. Freire,[b] Érica L. Santos,[b] Flávio M. Alves,[a] Ana C. Micheletti,[b] and Nídia C. Yoshida*,[b]

[a] Instituto de Biociências, Universidade Federal de Mato Grosso do Sul, 79070-900 Campo Grande, MS, Brazil

[b] Instituto de Química, Universidade Federal de Mato Grosso do Sul, 79074-460 Campo Grande, MS, Brazil (e-mail*: nidia.yoshida@ufms.br)

LIST OF CONTENTS

Figure 1. Structure of compound **13** isolated from the dichloromethane phase of the *Mezilaurus duckei* leaf extract.

Table 1. ^{1}H (300 MHz) and ^{13}C (75 MHz) NMR spectral data ($CDCl_3$) of compound **13**, compared with rubrenolide (Tófoli et al., 2016).

Figure 2. ^{1}H NMR (300 MHz, $CDCl_3$) spectrum of compound **13** (rubrenolide) isolated from the dichloromethane phase of the *Mezilaurus duckei* leaf extract.

Figure 3. ^{13}C NMR (75 MHz, $CDCl_3$) spectrum of compound **13** (rubenolide), isolated from fraction F of the dichloromethane phase of the *Mezilaurus duckei* leaf extract.

Figure 4. Structure of compound **8** isolated from fraction M of the dichloromethane phase of the *Mezilaurus duckei* leaf extract

Table 2. ^{1}H NMR (300 MHz, acetone-d_6) data of compound **8**, compared with kaempferol (Itoh et al., 2009).

Figure 5. ^{1}H NMR (300 MHz, acetone-d_6) spectrum (with expansion) of compound **8** (kaempferol) from fraction M of the dichloromethane phase of the *Mezilaurus duckei* leaf extract.

Figure 1. Structure of compound **13** isolated from the dichloromethane phase of the *Mezilaurus duckei* leaf extract.

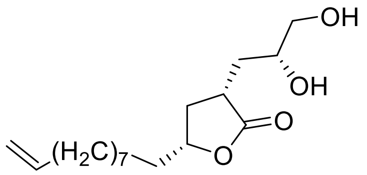

Rubrenolide (**13**)

Table 1. ^1H (300 MHz) and ^{13}C (75 MHz) NMR spectral data ($CDCl_3$) of compound **13**, compared with rubrenolide (Tófoli et al., 2016).

Position	δ^{1}H of Compound 13 ($CDCl_3$) (J in Hz)	δ^{1}H Rubrenolide ($CDCl_3$)[a] (J in Hz)	δ^{13}C of Compound 13 ($CDCl_3$)	δ^{13}C Rubrenolide ($CDCl_3$)[a]
**1**	–	–	180.4	180.0
**2**	2.83–2.94 m	2.83–2.94 m	38.8	38.7
**3**	2.53 ddd (12.3, 8.4, 5.4), 1.54–1.62 m	2.53 ddd (12.3, 8.4, 5.4), 1.54–1.62 m	35.7	35.7
**4**	4.31–4.42 m	4.31–4.42 m	79.8	79.9
**5**	1.71–1.81 m, 1.56–1.66 m	1.71–1.81 m, 1.56–1.66 m	35.3	35.3
**6**	1.42–1.52 m, 1.32–1.42 m	1.42–1.52 m, 1.32–1.42 m	25.1	25.2
**7**	1.25–1.40 sl	1.25–1.40 sl	29.0	29.3
**8**	1.25–1.40 sl	1.25–1.40 sl	29.0	29.0
**9**	1.25–1.40 sl	1.25–1.40 sl	29.0	29.3
**10**	1.25–1.40 m	1.25–1.40 m	29.0	28.8
**11**	1.25–1.40 sl	1.25–1.40 sl	29.0	29.3
**12**	2.00 dd (14.0, 6.6)	2.00 dd (14.0, 6.6)	33.7	33.7
**13**	5.75 ddt (16.8, 11.2, 6.6)	5.75 ddt (16.8, 11.2, 6.6)	139.0	139.1
**14**	4.95 br d (16.8), 4.89 br d (11.2)	4.95 br d (16.8), 4.89 br d (11.2)	114.1	114.1
**1′**	1.90–2.00 m, 1.55–1.61 m	1.90–2.00 m, 1.55–1.61 m	33.7	33.7
**2′**	3.65–3.75 m	3.65–3.75 m	70.3	70.2
**3′**	3.59 br d (10.8), 3.44 dd (10.8, 6.3)	3.59 br d (10.8), 3.44 dd (10.8, 6.3)	66.6	66.6

[a)] ^1H NMR spectra recorded at 500 MHz and ^{13}C NMR spectra at 125 MHz (Tófoli et al., 2016).

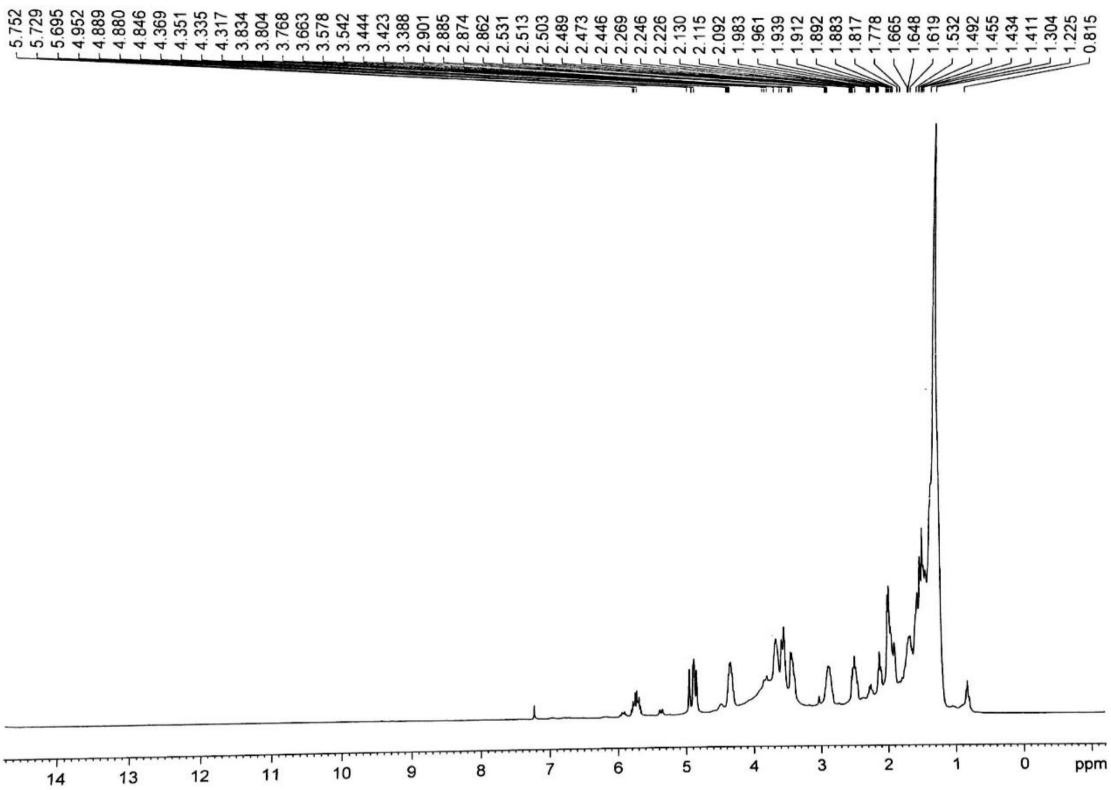

Figure 2. ¹H NMR (300 MHz, $CDCl_3$) spectrum of compound **13** (rubrenolide) isolated from the dichloromethane phase of the *Mezilaurus duckei* leaf extract.

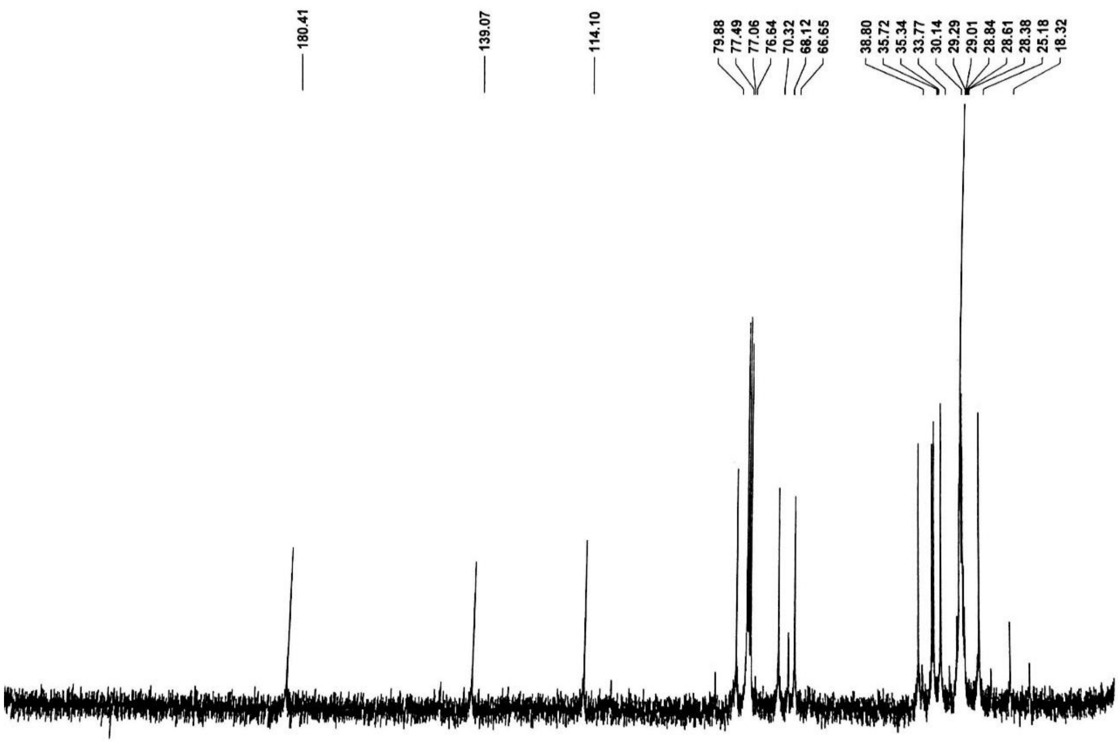

Figure 3. ¹³C NMR (75 MHz, $CDCl_3$) spectrum of compound **13** (rubenolide), isolated from fraction F of the dichloromethane phase of the *Mezilaurus duckei* leaf extract.

Figure 4. Structure of compound **8** isolated from fraction M of the dichloromethane phase of the *Mezilaurus duckei* leaf extract

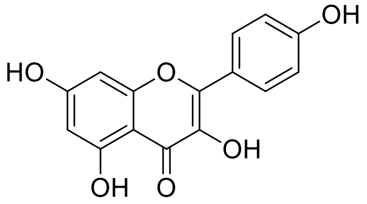

Kaempferol (**8**)

Table 2. ^{1}H NMR (300 MHz, acetone-d_6) data of compound **8**, compared with kaempferol (Itoh et al., 2009).

**Position**	**δ^{1}H of Compound 8 ($CDCl_3$) (J in Hz)**	**Kaempferol^b $δ_H$**
**2**	-	-
**3**	-	-
**4**	-	-
**5**	-	-
**6**	6.26 d (J=1.8)	6.27 d (J=2.0)
**7**	-	-
**8**	6.53 d (J=1.8)	6.34 d (J=2.0)
**9**	-	-
**10**	-	-
**1'**	-	-
**2'**	8.14 *d* (*J*=8.5)	7.98 d (J=8.3)
**3'**	7.00 *d* (*J*=8.5)	6.96 *d* (*J*=8.3)
**4'**	-	-
**5'**	7.00 *d* (J=8.5)	6.96 *d* (*J*=8.3)
**6'**	8.14 *d* (J=8.5)	7.98 d (J=8.3)
**OH**	12.18 s	-

b) ^{1}H NMR spectra recorded at 600 MHz (Itoh et al., 2009).

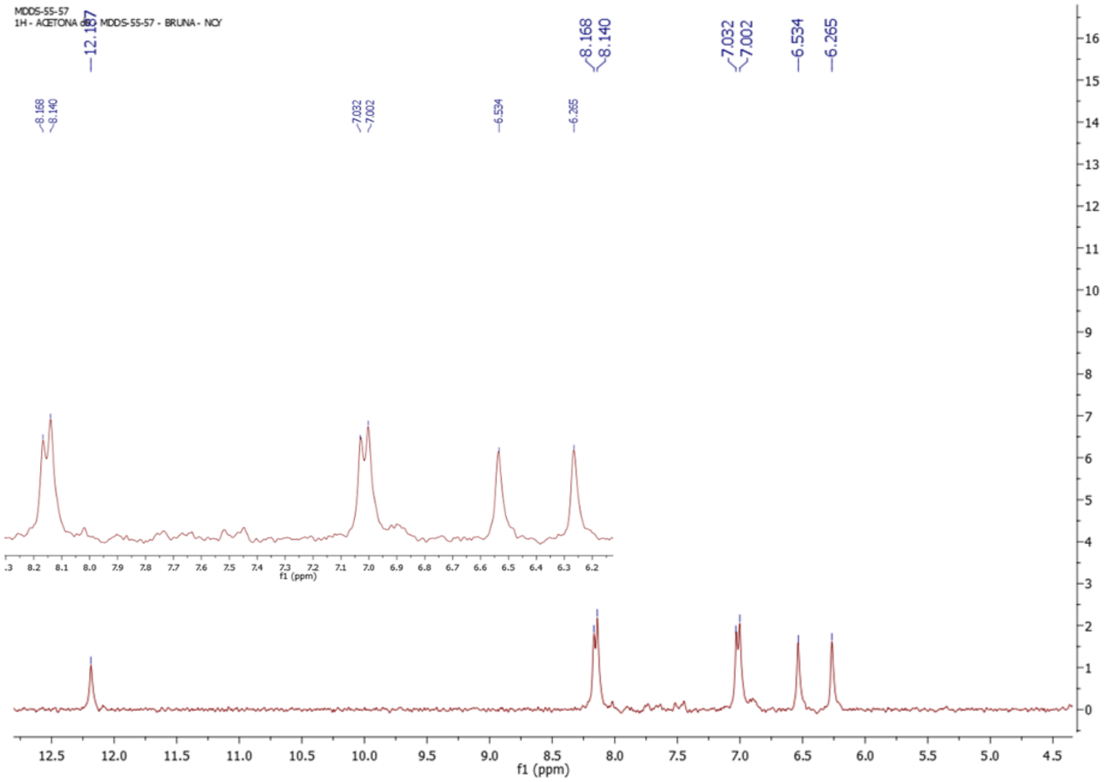

Figure 5. ^{1}H NMR (300 MHz, acetone-d_6) spectrum (with expansion) of compound **8** (kaempferol) from fraction M of the dichloromethane phase of the *Mezilaurus duckei* leaf extract.

REFERENCES

Tófoli, D., Martins, L. A. V., Matos, M. F. C., Garcez, W. S., & Garcez, F. R. (2016). Antiproliferative butyrolactones from *Mezilaurus crassiramea*. *Planta Medica Letters, 3*(1), e14–e16.

Itoh, T., Ninomiya, M., Yasuda, M., Koshikawa, K., Deyashiki, Y., Nozawa, Y., Akao, Y., & Koketsu, M. (2009). Inhibitory effects of flavonoids isolated from *Fragaria ananassa* Duch on IgE-mediated degranulation in rat basophilic leukemia RBL-2H3. *Bioorganic & Medicinal Chemistry, 17*(15), 5374–5379.
